# Determine TB-LAM lateral flow urine antigen assay for HIV-associated tuberculosis: recommendations on the design and reporting of clinical studies

**DOI:** 10.1186/1471-2334-13-407

**Published:** 2013-09-03

**Authors:** Stephen D Lawn, Keertan Dheda, Andrew D Kerkhoff, Jonathan G Peter, Susan Dorman, Catharina C Boehme, Mark P Nicol

**Affiliations:** 1 Department of Clinical Research, Faculty of Infectious and Tropical Diseases, London School of Hygiene and Tropical Medicine, Keppel Street, London WC1E 7HT, UK; 2 The Desmond Tutu HIV Centre, Institute for Infectious Disease and Molecular Medicine, Faculty of Health Sciences, University of Cape Town, Cape Town, South Africa; 3 Lung Infection and Immunity Unit, Division of Pulmonology & UCT Lung Institute, Department of Medicine, University of Cape Town, Cape Town, South Africa; 4 George Washington University School of Medicine and Health Sciences, Washington, DC, USA; 5 TB vaccine group, Jenner Institute, University of Oxford, Oxford, UK; 6 Johns Hopkins University School of Medicine, Baltimore, MD, USA; 7 Foundation for Innovative New Diagnostics (FIND), Geneva, Switzerland; 8 Division of Medical Microbiology, Faculty of Health Sciences, University of Cape Town, Cape Town, South Africa; 9 National Health Laboratory Service, Groote Schuur Hospital, Cape Town, South Africa

**Keywords:** Determine TB-LAM Ag, Lipoarabinomannan, Tuberculosis, Diagnosis, Diagnostic accuracy, Sensitivity, Specificity, Point-of-care

## Abstract

Detection of the *Mycobacterium tuberculosis* cell wall antigen lipoarabinomannan (LAM) in urine permits diagnoses of tuberculosis (TB) to be made in HIV-infected patients with advanced immunodeficiency. This can be achieved at the point-of-care within just 30 minutes using the Determine TB-LAM, which is a commercially available, lateral-flow urine ‘strip test’ assay. The assay has been shown to have useful diagnostic accuracy in patients enrolling in antiretroviral treatment services or in HIV-infected patients requiring admission to hospital medical wards in sub-Saharan Africa. Such patients have high mortality risk and have most to gain from rapid diagnosis of TB and immediate initiation of treatment. However, few studies using this assay have yet been reported and many questions remain concerning the correct use of the assay, interpretation of results, the role of the assay as an add-on test within existing diagnostic algorithms and the types of further studies needed. In this paper we address a series of questions with the aim of informing the design, conduct and interpretation of future studies. Specifically, we clarify which clinical populations are most likely to derive benefit from use of this assay and how patients enrolled in such studies might best be characterised. We describe the importance of employing a rigorous microbiological diagnostic reference standard in studies of diagnostic accuracy and discuss issues surrounding the specificity of the assay in different geographical areas and potential cross-reactivity with non-tuberculous mycobacteria and other organisms. We highlight the importance of careful procedures for urine collection and storage and the critical issue of how to read and interpret the test strips. Finally, we consider how the assay could be used in combination with other assays and outline the types of studies that are required to build the evidence base concerning its use.

## Background

HIV-associated tuberculosis (TB) remains a major challenge to global health with an estimated 1.1 million new cases and 430,000 deaths in 2011 [1]. A majority of cases (79%) are in sub-Saharan Africa where health infrastructure and laboratory services are least well developed to tackle this challenge. Post-mortem studies conducted among hospital in-patients who died with HIV/AIDS in Africa have reported the presence of TB (often disseminated) in between 30% and 50% of cadavers [2-5]. Much of this disease remained undiagnosed at the time of death, highlighting the enormous challenge of TB screening and diagnosis in those with HIV infection. However, in recent years, new assays have been developed and evaluated and have been demonstrated to have utility among HIV-infected patients. These include the Xpert MTB/RIF automated rapid molecular assay (Cepheid Inc., Sunnyvale, CA, USA) [6,7] and the Determine TB-LAM (Alere Inc., Waltham, MA, USA) lateral flow urine antigen test [8].

The Xpert MTB/RIF assay, which was endorsed by the World Health Organization (WHO) in 2010, has much greater sensitivity than sputum smear microscopy in HIV-infected patient populations and is now being increasingly implemented in many resource-limited countries [7]. However, while this is undoubtedly a landmark development in TB diagnostics, its relatively high cost and the need for sophisticated instrumentation, reliable power supply and linkage to a computer will limit its use. Unfortunately, it does not fulfil many of the ideal characteristics of a point-of-care test and its use will likely be confined largely to the laboratory environment [7]. Separation of testing from the clinical interface may undermine potential impact on clinical outcomes since the scope for same-day decision making regarding initiation of TB treatment is limited [9,10]. This may be particularly problematic in settings with a high rate of initial default and especially among HIV-infected patients with advanced immunodeficiency in whom delays in diagnosis may be associated with considerable mortality risk [11].

The Determine TB-LAM assay diagnoses TB by detecting the presence of the *Mycobacterium tuberculosis* cell wall antigen lipoarabinomannan (LAM) in urine of HIV-infected patients [8]. Despite having lower sensitivity compared to the Xpert MTB/RIF assay [12], the main advantages of the Determine TB-LAM test are its simplicity of use, lack of instrumentation, speed of use with results available after 25 minutes, low cost (initially marketed at $3.50 per test) and the fact that it has the potential to be implemented at the point-of-care. These characteristics make this potentially a very useful ‘add-on test’ within the TB diagnostic algorithm. Shortened time to initiation of treatment in patients with HIV-associated TB reduces mortality risk [13] and rapid point-of-care diagnostics are likely to be the best means of achieving this.

Earlier studies evaluating the enzyme-linked immunosorbent assay (ELISA) format of the urine LAM assay have previously been summarized and reviewed [8,14] but far fewer data are available regarding the use of the Determine TB-LAM lateral flow version of the assay. Currently available data on the diagnostic accuracy of Determine TB-LAM from published studies or recent conference proceedings are summarized in the Table 1. These data largely confirm that the sensitivity of Determine TB-LAM is greatest among HIV-infected patients with the most advanced immunodeficiency. However, there is considerable heterogeneity in the data from studies of both the ELISA and lateral flow versions of the assay. This may relate to differences in the patient populations studied or to methodological or technical issues which are discussed later in this article. These issues require careful consideration when interpreting results and must be taken into account when designing further studies.

**Table 1:** Studies reporting on the diagnostic accuracy of the determine TB-LAM assay

**Study**	**Location**	**Patient population**	**No. of**	**Median CD4**	**Number of **	**Reference**	**Urine testing**	**Overall**	**Sensitivity by**	**Overall**	**Results by**
			**patients**	**count (IQR)**	**TB cases**	**standard**	**(retrospective**	**sensitivity**	**CD4 count**	**specificity**	**grade 1**
			**screened**		**(PTB/EPTB)**		**or prospective)**	**n/N (%)**	**n/N (%)**	**n/N (%)**	**or grade 2**
											**cutoff**
Lawn et al. (2012) [7]	Cape Town, South Africa	Active screening of HIV + outpatients pre-ART	516	170 (100–233)	PTB = 85	Sputum culture (liquid)	Retrospective	24/85 (28.2)	<50: 12/18 (66.7)	425/431 (98.6)	Grade 1
									<100: 15/29 (51.7)		
									<200: 23/59 (39.0)		
									≥200: 1/25 (4.0)		
Peter et al. (2012) [15]	Cape Town, South Africa	Inpatient HIV + TB suspects	281 TB suspects +88 non-TB controls	90 (47–197)	PTB + EPTB = 116	Sputum and non-sputum culture (liquid)	Retrospective	Grade 1 cutoff: 77/116 (66)	Grade 1 cutoff:	Grade 1 cutoff: 79/88 (89.8)^†^	Grade 1 and 2 reported
									≤200: 58/81 (72) >200: 14/26 (54)		
								Grade 2 cutoff: 58/116 (50)	Grade 2 cutoff:	Grade 2 cutoff: 87/88 (98.9)^†^	
									≤200: 47/81 (58) >200: 7/26 (27)		
Dorman et al. (2012) [16]	Cape Town, South Africa and Kampala, Uganda	Inpatient and outpatient HIV + TB suspects	997	152 (41-337)	Total cases = 16	Sputum culture (solid and liquid), + mycobacterial blood culture	Prospective	136/367 (37.1)	≤100: 116/196 (59.2)	559/573 (97.6)	Grade 2
					PTB = 243						
					PTB + mycobact-eremia = 108						
									>100: 20/169 (11.8)		
					Mycobacteremia alone = 16						
Van Rie et al. (2013) [17]	Johannesburg, South Africa	Mostly inpatient HIV + disseminated TB and EPTB suspects who could not produce sputum sample or were sputum smear and sputum Xpert negative (17% of patients NOT HIV+)	219	116 (34–221)	EPTB + disseminated = 51	Culture (sputum, blood, lymph node FNA, CSF pleural fluid, ascitic fluid, urine)	Not stated	Any positive culture- 35/51 (68.8)	In HIV-positives:	Any positive culture: (91.8)	Grade 2
									<100: 82.6%		
									100–199: 63.3%		
									≥200: 40.0%		
									In HIV negatives: 0%		
Drain et al. (2013) [18]	Durban, South Africa	Newly diagnosed HIV + out-patients	342	180 (73–311)	60 PTB (positive microbiology)	Sputum smear or culture or Positive microbiology + clinical diagnoses	Prospective	PTB (micro+) only: 17/60 (28.3)	PTB (micro+) only:	No micro + PTB diagnosis: 254/282 (90.1)	Unkown
									<100: 12/32 (37.5)		
									100-199: 3/12 (25.0)		
									200-349: 1/3 (33.3)		
									≥350: 1/5 (20.0)		
					99 including PTB + EPTB + clinical diagnoses			All diagnoses: 24/99 (24.2)	All diagnoses	No TB diagnosis: 222/243 (91.2%)	
									<100: 14/50 (28.0)		
									100-199: 4/20 (20.0)		
									200-349: 2/6 (33.3)		
									≥ 350: 2/11 (18.2)		
Shah et al. (2013) [19]	Uganda	Inpatient and outpatient HIV + TB suspects	101 TB cases + 105 TB suspects with TB excluded	60 among those with TB	103 PTB + EPTB	Sputum culture (solid and liquid), mycobacterial blood culture	Retrospective	50/103 (48.5)	<50:30/46 (65.2)	102/105 (97.1)	Grade 2
									51-100: 12/17 (70.6)		
									101-200: 4/19 (21.1)		
									>200: 4/21 (19.0)		

^†^Specificity reported is for a control group of non-TB suspects to illustrate differences between grade 1 and 2 cut-offs.

Following its commercial launch in January 2013, Determine TB-LAM remains the focus of ongoing clinical evaluation studies. As the evidence base grows, data on this assay may be reviewed by an expert panel convened by the WHO – potentially in 2014. Below we address key issues that need to be carefully considered in further evaluations of this assay which we present as a series of questions. We hope that this paper will help to inform the design, conduct and interpretation of future clinical studies and accelerate the expert review process. The opinions expressed in this article are those of the individuals represented in the authorship and are based upon the evidence base available at the time of writing. Many of the data referred to are from studies conducted by the authors, providing a perspective of those working directly in this field of research.

## Main text

### 1. Which patient populations should be prioritized for further study of Determine TB-LAM?

Current evidence strongly indicates that the urine LAM antigen assays only have useful diagnostic accuracy among HIV-infected adult patients and should not be used to investigate those who are HIV-negative [14]. There are currently no published data from studies in children. In addition, assay sensitivity is strongly related to the degree of immunodeficiency, with graded increases in sensitivity with diminishing CD4 counts. Current evidence shows that the ELISA and the lateral flow formats of the assay do not have useful diagnostic accuracy among patients with CD4 counts greater than approximately 200 cells/μL or among those who have early clinical stages of disease (WHO stages 1 or 2) prior to TB diagnosis. Thus, the specific clinical populations in which the assay might be useful include hospital in-patients with known HIV infection [15] and ambulatory out-patients with known HIV-infection and low CD4 cell counts such as those newly referred to antiretroviral treatment (ART) clinics [12]. The assay should not be used to screen or investigate unselected patients in primary care as the positive predictive value is very limited among those who are either HIV-infected and have high CD4 counts or who are HIV-negative.

### 2. What patient characterization helps interpretation of study data?

In addition to HIV status and CD4 cell count, the sensitivity of Determine TB-LAM varies substantially according to disease severity as reflected by a range of other prognostic indices [16,20-23]. Thus, careful characterization of the study population may help with data interpretation and with assessment of the external validity of the data. In general, the sicker patients are, the greater the sensitivity of Determine TB-LAM. LAM-positive TB is associated with more advanced WHO stages of disease and higher Modified Early Warning Scores (MEWS), which are both simple clinical assessments of disease severity [21]. Higher sensitivity is found in hospital in-patients compared to ambulatory out-patients [16] and sensitivity is positively correlated with serum concentrations of serum C-reactive protein (CRP) [22], which is a strong prognostic marker [20,24]. Higher sensitivity is also strongly associated with increasing severity of anaemia and with more advanced symptoms in patients with HIV-associated TB (Lawn SD, unpublished data). Thus, careful characterisation of patient populations using parameters such as symptom profile, CD4 cell count, blood haemoglobin, body mass index, C-reactive protein as well as simple validated illness severity and morbidity tools such as MEWS or APACHE scores in studies of in-patients may help with the interpretation and comparison of study data in future studies.

### 3. What is the appropriate reference standard in studies of diagnostic accuracy?

Obtaining appropriate clinical samples and the use of optimum microbiological methods to provide a rigorous reference standard is a critical issue. Most existing studies evaluating the diagnostic accuracy of LAM assays have used sputum culture alone as the microbiological reference standard. This approach is potentially problematic as false-negative reference standards could arise in patients in whom it is difficult to obtain sufficiently high quality sputum samples, in those with HIV-associated TB who do not have pulmonary involvement and also due to variation in sputum processing protocols, including stringency of sputum decontamination procedures. These issues are likely to be particularly important in HIV-infected patient populations as bacillary load in sputum is typically low. Culture of multiple clinical samples including sampling from sites of disease for extrapulmonary TB will enhance the reference standard. These should be tested in quality-assured laboratories using liquid culture which has the highest sensitivity for *Mycobacterium tuberculosis* of any assay.

Sputum samples should be carefully obtained (this might be aided by sputum induction using nebulized hypertonic saline [25,26]) and more than one sputum sample should ideally be tested in light of the significant incremental yield. Simple patient instruction by healthcare workers on how to correctly expectorate sputum is also an important intervention to improve sample quality, especially in settings without sputum induction facilities. When compared with sputum induction in a primary care setting, it was found to offer equivalent diagnostic yields with lower cost and fewer adverse events when investigating initially smear-negative or sputum-scarce TB patients using smear microscopy or Xpert MTB/RIF to test additional sputum samples [27]. Additional extrapulmonary samples such mycobacterial blood cultures [28] or fine needle lymph node aspirates [29,30] might also be obtained for culture or Xpert MTB/RIF testing. Moreover, testing of urine using Xpert MTB/RIF or culture may provide a further logical addition to the reference standard [31,32]. Although LAM-antigenuria has been found to co-exist with mycobacteriuria in a substantial proportion of patients [31,33], antigenuria may also be present in urine testing negative with Xpert MTB/RIF. Thus, simply testing urine for the presence of *M. tuberculosis* cannot be used as the sole reference standard.

A comprehensive microbiological assessment is the ideal reference standard as this excludes the considerable uncertainties that inevitably surround clinical and radiological diagnoses. But in practice, obtaining multiple high quality samples on large cohorts of patients is challenging and very expensive. Clinical and radiological assessments are often recorded, but reliance on non-microbiologically confirmed TB diagnoses and responses to TB treatment is a less robust approach. Patients with bacterial sepsis such as pneumococcal pneumonia, for example, may respond to rifampicin-containing TB treatment and improvements in clinical condition may be confounded by other factors such as the initiation of ART. Moreover, prospective follow-up may be complicated by new onset incident TB.

### 4. Do non-tuberculous mycobacteria cause false-positive results?

LAM encompasses a large family of related molecules which are expressed by mycobacterial species and so cross-reactivity between the assay antibodies and non-tuberculous mycobacterial antigen is possible [8]. This is therefore a potential source of diminished assay specificity. Although a small number of patients have been reported in studies as having false-positive urine LAM results and have had non-tuberculous mycobacteria cultured from sputum samples, it is not clear whether these organisms were true pathogens or sputum contaminants [8]. Studies in which detection of LAM has been assessed in patients with confirmed non-tuberculous mycobacterial pulmonary or disseminated disease are lacking. Future studies of the diagnostic accuracy of Determine TB-LAM studies should ideally report on the species of the cultured isolates of non-tuberculous mycobacteria and carefully seek to determine the clinical significance of these isolates.

### 5. Is high assay specificity limited to studies in South Africa?

Many of the studies on TB diagnosis using urine LAM assays have been done in South Africa with most others being from other southern and east African countries. Comparable studies using the LAM ELISA format of the assay done within South Africa found specificity to lie in the range 96% to 100% [12,34-37] whereas several studies from Tanzania, Zimbabwe and India reported specificities of 88% - 89% [38-40]. The reasons for this have not been conclusively defined. However, it has been speculated that a range of factors that might have contributed to lower observed specificity in the latter studies might include the use of solid rather than liquid culture as the reference standard, investigation of predominantly HIV-uninfected patients with a lower pre-test probability of TB (an ‘off-label’ indication for the assay), use of non-sterile containers to store urine and the higher frequency of cultures of non-tuberculous mycobacteria [8]. An important observation from a multi-country study using standardized methodology was that the specificity of Determine TB-LAM did not differ between South African and Ugandan study sites [16], which provides important reassurance about assay performance in different geographical settings. However, many more studies are needed in different countries and regions.

### 6. How should urine samples be collected and stored?

Anti-LAM antibodies used in the commercial LAM assays cross-react with a number of other bacteria, including those present in oral flora such as various species of Actinobacteria (Nocardia and Streptomyces), Candida [36] and non-tuberculous mycobacteria [8]. Cross-reactive bacteria from the perineum or in faecal material could contaminate urine samples as might environmental organisms present in non-sterile urine collection disposables and containers. Obtaining urine samples in studies of very young children may be particularly challenging. The presence of such organisms is likely to become increasingly important if samples are left at room temperature for prolonged periods, permitting microbial replication. Thus, collection of a clean sample should be done following careful instruction to the patient. Whether this should be a mid-stream sample has yet to be defined but sterile containers should be used. Some data suggest that concentrations of LAM spiked into urine samples are stable for the first 2 hours but that they deteriorate after this [41]. One of the authors (SDL) has found considerable loss of LAM reactivity of urine samples stored for 3 years at −20°C and following at least 2 freeze-thaw cycles (unpublished data). Thus, we recommend that samples be tested immediately. If this is not possible, samples should be stored by freezing as soon as possible following sample collection and that freeze-thaw cycles are minimized. A series of carefully conducted analytic studies is needed to address these issues.

### 7. How should determine TB-LAM test strips be interpreted?

Interpretation of lateral-flow assays designed to provide a binary (positive / negative) result can be challenging [42]. The Determine TB-LAM product insert accompanying the assay at the time of its commercial launch in 2013 did not provide clear guidance regarding how to precisely interpret the test strip results. The assay includes a reference card with bands of graded intensity to facilitate the reading of results. In the literature, one research group has referred to the least intense band as the ‘grade 1 cut-point’ and the band of next intensity has been termed the ‘grade 2 cut-point’ [15]. In the rest of this article we have adopted similar terminology.

In three separate studies conducted by the authors of the present article, investigators scored samples as positive at the grade 1 cut-point in a similar way to each other. In these studies, a positive result was defined as a band of equal or greater intensity as the grade 1 cut-point. Visible bands that were discernible but were weaker in intensity than the grade 1 cut-point were scored as negative. In two of the studies, results were also interpreted using the grade 2 cut-point, but the two research groups used slightly different definitions. Peter and colleagues defined a positive result at the grade 2 cut-point by the presence of a band of at least equal intensity to the grade 2 reference band. In contrast, Dorman and colleagues scored a positive result at this cut-point as being the presence of a band whose intensity was greater than that of a grade 1 band but closer in intensity to a grade 2 than a grade 1 band. Clearly, careful standardization of the reading methodology is a critical need.

Using the grade 1 cut-point and a rigorous reference standard of liquid culture on two sputum samples (spot plus induced), Lawn and colleagues studying ambulatory patients screened prior to starting ART found very high specificity (98.6%; 95%CI, 97.0-99.5). Interim analysis of data from a follow-on study among in-patients found specificity a little lower using the grade 1 cut-point but approximately 99% using the grade 2 cut-point [43]. In contrast, both studies by Peter and colleagues and Dorman and colleagues found much more limited specificity using the grade 1 cut-point and that the area under the receiver operator characteristic (ROC) curve was greater using a grade 2 cut-point (Table 1). Thus, switching from a grade 1 to a grade 2 cut-point increased specificity substantially but was also associated with some loss of sensitivity in a study from South Africa (specificity, 90% vs. 99%, p = 0.009; sensitivity: 60% vs. 45%, p < 0.001) [15]. These differences were even more marked in the study conducted in Uganda and South Africa in which the sensitivity of grade 1 and grade 2 cut-points were 61.6% and 37.1%, respectively, and the specificities were 78.4% and 97.6%, respectively [16].

The apparent discrepancies between the optimum cut-off in these studies may relate to a number of factors including differences in precisely how the strips were interpreted, the reference standard used and differences in patient populations. However, all investigators of these three studies are in agreement that reading the strips at the grade 1 cut-point is challenging and takes a great degree of care that is unlikely to be a realistic expectation of non-laboratory trained health-care workers in the busy clinic environment. Thus, the authors suggest that use of the grade 2 cut-point is more likely to provide a reproducible test result and be feasible to use in clinical practice in the clinic environment. Future studies of both diagnostic accuracy and operational feasibility should evaluate both cut-offs to increase the evidence base. The method used by Peter et al. would represent the simplest approach and would potentially permit results to be read using visual comparison with a revised version of the reference card displaying a single (grade 2 intensity) standard band. Positive results would then simply be defined by a test band of equal or greater intensity as the single reference band.

### 8. Can this be used as a stand-alone test?

It is clear that Determine TB-LAM cannot be used as a stand-alone test but has a potentially important role as an add-on test within the diagnostic algorithm. Its limited sensitivity requires that it is used in conjunction with other diagnostic tests that collectively provide adequate sensitivity for TB diagnosis as well as sufficiently high negative predictive value to rule out TB. However, data are emerging that Determine TB-LAM not only provides the most rapid means of conducting an initial diagnostic screen at the point-of-care, but also yields incremental diagnostic sensitivity when combined with sputum microscopy [12,15] and Xpert MTB/RIF testing of sputum [16]. Moreover, it has high positive predictive value among patients with abnormal chest radiographs [12]. Studies need to define how to best use Determine TB-LAM in combination with other assays within the diagnostic algorithm to optimise diagnostic accuracy, minimize time to diagnosis, reduce initial treatment default and mortality and to provide the most efficient and cost-effective diagnostic process.

### 9. What types of studies on determine TB-LAM are needed?

To date, most studies of Determine TB-LAM have assessed diagnostic accuracy, with testing of stored urine samples being done retrospectively. A series of coordinated studies are now needed to:

i. increase the evidence base for the diagnostic accuracy in the appropriate clinical populations;

ii. define the optimum methods of urine collection to minimise the likelihood of urine contamination (and optimimum storage if later retrospective analyses are also to be done).

iii. assess the operational feasibility of prospective use of the assay in the clinical environment;

iv. define at which levels of the health care system the assay can be used and by which cadres of health care workers;

v. define how to incorporate the assay into the diagnostic algorithm;

vi. assess the impact of implementation of the assay on clinical outcomes by means of randomised controlled trials (one such trial is in progress: http://clinicaltrials.gov/show/NCT01770730;

vii. assess cost-effectiveness in different settings and algorithms.

viii. assess operational feasibility of use by national TB control programmes, including systems for reporting and surveillance.

## Conclusion

The Determine TB-LAM assay offers the prospect of low-cost point-of-care diagnosis of HIV-associated TB among patients with advanced HIV-associated immunodeficiency in whom mortality risk is greatest. Future studies on the use of this as an add-on assay within diagnostic algorthims require careful design and appropriate standardization to build a strong evidence base for implementation.

## Abbreviations

AIDS: Acquired immune deficiency syndrome; ART: Antiretroviral therapy; HIV: Human immunodeficiency virus; LAM: Lipoarabinomannan; TB: Tuberculosis; WHO: World Health Organization.

## Competing interests

SDL, SED, MN, JP and KD have received donations of Determine TB-LAM strips from the manufacturer (Alere Inc) in previous studies as unconditional gifts. Alere has taken no part in the design, conduct or interpretation of these studies and has not contributed to the content of this or other manuscripts arising from these studies. The authors declare that they have no competing interests.

## Authors’ contributions

SDL initiated and coordinated the writing of this paper and wrote the first draft. All authors contributed to subsequent drafts and approved the final version. All authors read and approved the final manuscript.

## Pre-publication history

The pre-publication history for this paper can be accessed here:

http://www.biomedcentral.com/1471-2334/13/407/prepub
